# Early dynamic behavior of lactate in predicting continuous renal replacement therapy after surgery for acute type A aortic dissection

**DOI:** 10.3389/fcvm.2022.948672

**Published:** 2022-07-26

**Authors:** Zhigang Wang, Jingfang Xu, Yubei Kang, Ling Liu, Lifang Zhang, Dongjin Wang

**Affiliations:** ^1^Department of Cardio-Thoracic Surgery, Affiliated Drum Tower Hospital, Medical School of Nanjing University, Nanjing, China; ^2^Department of Nephrology, Nanjing Drum Tower Hospital Clinical College of Nanjing University of Chinese Medicine, Nanjing, China; ^3^Department of Psychiatry, The First Affiliated Hospital, Zhengzhou University, Zhengzhou, China

**Keywords:** aortic dissection, continuous renal replacement therapy, perioperative lactate, lactate clearance, risk factor

## Abstract

**Background:**

It has been well known that hyperlactatemia is an independent risk factor for postoperative mortality in patients who received acute type A aortic dissection (ATAAD) surgery. Some patients may require the assistance of continuous renal replacement therapy (CRRT) for acute postoperative renal deficiency and often associate with increased mortality rate. This study aimed to examine the association between the early dynamic change of lactate levels and postoperative CRRT in ATAAD patients who received surgical repairment.

**Methods:**

This retrospective study included 503 patients who received ATAAD surgeries. Serum lactate levels were measured before operation and at 0, 1, 3, 6, 12, 24 h post intensive care unit (ICU) admission. We examined the association between dynamic changes of lactate and CRRT.

**Results:**

Among all patients, 19.9% (100 patients) required CRRT. Our data showed that the lactate levels were higher in the CRRT group at all timepoints compared to the non-CRRT group. In a multivariate model, lactate levels at 12 h post ICU admission [odds ratio (OR), 1.362; *p* = 0.007] was identified as an independent predictor for requiring CRRT. Unsurprisingly, 30-day mortality in the CRRT group (41%) was 8.2 times higher than in the non-CRRT group (5%). To better understand the associations between CRRT and lactate levels, patients in the CRRT group were further stratified into the non-survivor group (*n* = 41) and survivor group (*n* = 59) based on the 30-day mortality. Elevated lactate levels measured upon ICU admission (OR, 1.284; *p* = 0.001) and decreased 24 h lactate clearance (OR, 0.237; *p* = 0.039) were independent risk factors for 30-day mortality in patients who received CRRT. The area under the curve to predict requirement for CRRT at 6 and 12 h post CICU admission were 0.714 and 0.722, respectively, corresponding to lactate cut-off levels of 4.15 and 2.45 mmol/L.

**Conclusion:**

The CRRT is commonly required in patients who received ATAAD surgery and often associated with worse mortality. Early dynamic changes of lactate levels can be used to predict the requirement of postoperative CRRT.

## Introduction

Despite recent advances in clinical management and surgical techniques, the prognosis of acute type A aortic dissection (ATAAD) remains unsatisfactory with 15–47% in-hospital mortality rates (1–4). Many risk factors for mortality after ATAAD repair have been identified including the use of continuous renal replacement therapy (CRRT) which is required in 15.9–22.4% of all ATAAD patients (5–7). Previous studies indicated that the mortality was as high as 40–80% among patients who received acute CRRT after ATAAD surgery (5, 8).

Lactate is mainly produced through pyruvate metabolism under anaerobic or aerobic glycolytic conditions and primarily removed by liver (9). It has been widely used in clinic as a surrogate marker to estimate tissue perfusion (10), and increasing levels of lactate associate with increased risks of death under infection, sepsis, and trauma conditions (11). The associations between lactate levels and increased morbidity as well as mortality have been well identified in cardiac surgery (12–16). A previous study suggested that patients with elevated lactate levels upon intensive care unit (ICU) admission (before CRRT initiation) were associated increased mortality rate after cardiac surgery (17). Compared to single measurement, recent reports conclude that serial lactate levels and lactate clearance are more reliable parameters to predict the risk (18, 19). Ludhmila et al. (14) found lactate level 6 h after ICU admission was an independent predictor of postoperative complications which included CRRT after cardiac surgery in adult patients. However, association between the dynamic changes of lactate levels and CRRT in patients who received ATAAD surgery has not been well studied. Current study not only aimed to describe the CRRT usage in a relatively large Chinese patient cohort but also explore its association with comprehensive lactate measurements.

## Materials and methods

### Patient data

**FIGURE 1:**
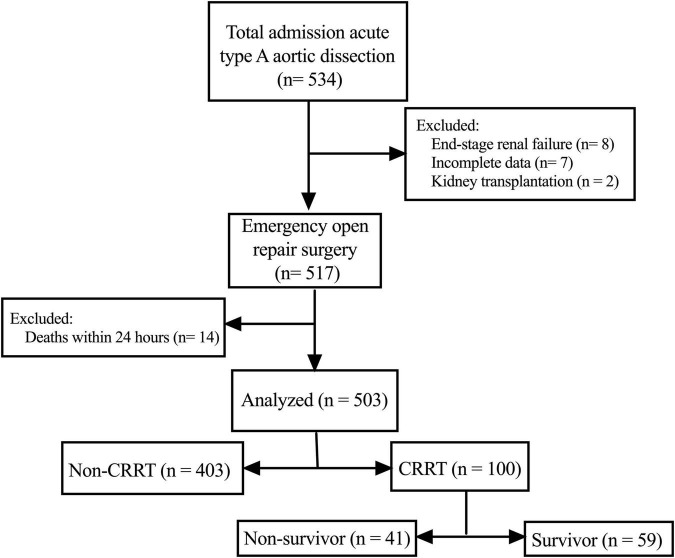
The patient selection process.

As shown in Figure 1, we retrospectively screened the electronic medical records and laboratory results of 534 patients who received ATAAD surgery at the Nanjing Drum Tower Hospital between January 2019 and December 2020. The ATAAD was diagnosed by enhanced computed tomography and within 14 days of symptom onset including intramural hematomas. Patients with following conditions were excluded from the study: (i) patients who received maintenance dialysis before surgery (*n* = 8); (ii) patients who received kidney transplantation (*n* = 2); (iii) patients with incomplete data (*n* = 7); (iv) patients who died within 24 h after surgery (*n* = 14). The Ethics Committee of Nanjing Drum Tower Hospital approved this study and waived the need for individual informed consent considering the retrospective nature of the study.

All patients were divided into the CRRT group and the non-CRRT group according to whether they received postoperative CRRT. Patients in the CRRT group were further stratified, based on the 30-day mortality, into survivors and non-survivors. All data including patient characteristics, laboratory results, postoperative complications or comorbidities, and outcomes were extracted from the electronic medical records system of our institution.

### Definitions

Acute kidney injury (AKI) was diagnosed based on the most recent Kidney Disease Improving Global Outcomes (KDIGO) criteria (20). Specifically, postoperative AKI was defined as worsening of the maximal change in the serum creatinine (sCr) concentration during the first postoperative week compared with preoperative baseline values. In this retrospective analysis, the urine output was not evaluated in the AKI diagnosis due to incomplete data. Hypotension was defined as systolic blood pressure less than 90 mmHg, regardless of the etiology. Prolonged mechanical ventilation (PMV) was defined as total intubation and ventilation time exceeded 48 h during the postoperative stay (21). The requirement for CRRT was determined by the severity of AKI (the sCr more than 354 μmol/L accompanied with the continuous decrease in urine output) and the overall conditions of patients.

### Lactate measurements

Blood lactate level was routinely measured upon hospital admission, at surgery and different time points after the operation (0, 1, 3, 6, 12, and 24 h after ICU admission). Addition measurements were also taken whenever it was necessary. Only arterial blood was collected to measure lactate levels (ABL Flex 800; Radiometer Medical, Copenhagen, Denmark).

Lactate clearance was calculated based on the level upon ICU admission with following equation: Lactate clearance (timepoint) = [lactate level upon ICU admission – lactate (time point)]/lactate level upon ICU admission.

### Statistical analysis

Data were analyzed with the SPSS for Windows version 26.0 (IBM Corp, Armonk, NY). Categorical variables were expressed as number and percentage and were analyzed using the chi-square or Fisher exact test as appropriate. Continuous variables were presented as mean ± standard deviation (or median and range) and compared with 2-tailed Student’s *t*-tests or Mann-Whitney *U*-tests, as appropriate. Normality was assessed with the Kolmogorov-Smirnov test.

A forward multiple logistic regression analysis was performed to identify independent predictive factors for receiving postoperative CRRT and 30-day mortality in patients with CRRT. This model included risk factors that were first identified by univariate analysis (*p* < 0.2). Hosmer and Lemeshow tests and −2 log likelihood provided an evaluation of the logistic regression model.

Receiver-operating characteristic (ROC) curves were constructed and the area under the ROC curve (AUC) was determined to estimate the accuracy of using variables of intent to predict the require for postoperative CRRT. The optimal cutoff value was assessed by You-den’s index (J = Sensitivity + Specificity – 1). All analyses were two-sided, and *p*-value of < 0.05 was considered statistically significant.

## Results

### Demographic and clinical characteristics

After screening, a total of 503 patients were eventually enrolled in this retrospective study. The demographic and clinical characteristics as well as laboratory results were summarized in Tables 1, 2. The mean age at surgery was 54.1 ± 13.6 years and the mean body mass index was 25.8 ± 3.9 kg/m^2^. Among all enrolled patients, 276 (54.8%) developed postoperative AKI and 100 (19.9%) required postoperative CRRT.

**TABLE 1:** Comparison of preoperative variables.

Variables	Total (*n* = 503)	CRRT (*n* = 100)	Non-CRRT (*n* = 403)	*P*-value
**Demographic data**				
Age (year)	54.1 ± 13.6	55.3 ± 13.6	53.8 ± 13.5	0.317
Male (%)	370 (73.6)	78 (78.0)	292 (72.5)	0.261
BMI (kg/m^2^)	25.8 ± 3.9	26.4 ± 4.5	25.7 ± 3.8	0.113
Smoking (%)	167 (33.2)	38 (38.0)	129 (32.0)	0.255
Drinking (%)	115 (22.9)	28 (28.0)	87 (21.6)	0.172
**Medical history**				
Hypertension (%)	439 (87.5)	92 (92.0)	347 (86.3)	0.125
Diabetes mellitus (%)	19 (3.8)	2 (2.0)	17 (4.2)	0.391
Previous cardiovascular disease (%)	15 (3.0)	4 (4.0)	11 (2.7)	0.512
Cerebrovascular disease (%)	20 (4.0)	4 (4.0)	16 (4.0)	1.000
Marfan syndrome (%)	9 (1.8)	0 (0)	9 (2.2)	0.216
COPD (%)	5 (1.0)	0 (0)	5 (1.2)	0.588
Atrial fibrillation (%)	9 (1.8)	3 (3.0)	6 (1.5)	0.392
**Previous cardiac surgery (%)**				
TEVAR (%)	10 (2.0)	3 (3.0)	7 (1.7)	0.425
CABG (%)	1 (0.2)	0 (0)	1 (0.2)	1.000
AVR (%)	2 (0.4)	0 (0)	2 (0.5)	1.000
Time from hospital to surgery (h)	4.0 (1.0, 9.5)	3.0 (1.0, 6.0)	4.0 (1.0, 9.0)	0.293
DeBakey type I (%)	405 (80.5)	87 (87.0)	318 (78.9)	0.067
Heart rate (bpm)	82.3 ± 40.6	82.6 ± 19.0	82.3 ± 44.4	0.952
**Presenting variables**				
Chest pain (%)	417 (82.9)	77 (77.0)	340 (84.4)	0.080
Back pain (%)	253 (50.3)	53 (53.0)	200 (49.6)	0.546
Abdominal pain (%)	50 (9.9)	14 (14.0)	36 (8.9)	0.130
Vomiting (%)	95 (18.9)	24 (24.0)	71 (17.6)	0.144
Limb ischemia (%)	69 (13.7)	17 (17.0)	52 (12.9)	0.286
Mesenteric ischemia (%)	12 (2.4)	7 (7.0)	5 (1.2)	**0.003**
Cerebral ischemia (%)	24 (4.8)	4 (4.0)	20 (5.0)	0.799
Coronary ischemia (%)	12 (2.4)	7 (7.0)	5 (1.2)	**0.003**
Involving renal artery (%)	191 (38.0)	42 (42.0)	149 (37.0)	0.354
Hypotension (%)	55 (10.9)	13 (13.0)	42 (10.4)	0.460
Pericardial effusion (%)	406 (81.9)	87 (87.9)	319 (80.4)	0.082

Values are mean ± standard deviation, median (interquartile range), or n (%).

BMI, body mass index; COPD, chronic obstructive pulmonary disease; TEVAR, thoracic endovascular aortic repair; CABG, coronary artery bypass graft; AVR, aortic valvule replacement.

Bold values represented p < 0.05.

**TABLE 2:** Comparison of laboratory tests upon admission.

Variables	Total (*n* = 503)	CRRT (*n* = 100)	Non-CRRT (*n* = 403)	*P*-value
WBC (10^9^/L)	11.2 ± 3.6	11.7 ± 3.7	11.0 ± 3.6	0.106
Hemoglobin (g/dL)	12.1 ± 2.5	11.9 ± 2.6	12.1 ± 2.5	0.553
Hematocrit (%)	34.2 ± 10.1	33.1 ± 11.2	34.5 ± 9.8	0.263
PLT (10^9^/L)	145.4 ± 71.9	135.9 ± 74.9	147.7 ± 71.0	0.146
Triglyceride (mmol/L)	1.2 ± 1.0	1.2 ± 1.1	1.2 ± 0.9	0.506
CRP (mg/dL)	74.6 ± 63.6	90.3 ± 70.9	70.7 ± 61.1	**0.006**
D-dimer (ng/mL)	6.1 (3.1, 11.8)	7.6 (3.8, 17.4)	5.6 (2.8, 10.9)	0.095
Albumin (g/L)	36.2 ± 6.7	35.7 ± 6.8	36.3 ± 6.6	0.402
TnT (ng/mL)	0.02 (0.01, 0.08)	0.02 (0.02, 0.11)	0.02 (0.01, 0.07)	0.111
CKMB (U/L)	10.0 (6.0, 15.0)	11.0 (7.0, 15.0)	10.0 (7.0, 15.0)	0.079
ALT (U/L)	19.0 (14.0, 44.0)	31.4 (14.9, 64.1)	18.5 (13.7, 40.9)	**0.025**
Bun (mmol/L)	8.9 ± 4.3	11.0 ± 5.6	8.3 ± 3.8	**<0.001**
sCr (μmol/L)	78.1 (61.0, 107.5)	101.4 (64.1, 144.3)	73.0 (59.3, 98.5)	**0.008**
BNP (pg/mL)	83.7 (31.3, 163.0)	61.3 (36.1, 187.0)	87.3 (32.9, 154.3)	0.181
Total bilirubin (mmol/L)	17.3 ± 14.2	18.1 ± 14.1	17.1 ± 14.2	0.553
PT (s)	13.1 ± 4.9	13.7 ± 3.4	13.0 ± 5.2	0.213
INR	1.2 ± 0.5	1.2 ± 0.2	1.2 ± 0.5	0.242
Fibrinogen (g/L)	2.1 ± 1.4	1.9 ± 1.1	2.2 ± 1.5	**0.035**

Values are mean ± standard deviation, median (interquartile range), or n (%).

WBC, white blood cell; PLT, platelet; CRP, c-reactive protein; TnT, troponin T; CKMB, creatine kinase myocardial band; ALT, alanine aminotransferase; Bun, blood urea nitrogen; sCr, serum creatinine; BNP, brain natriuretic peptide; PT, prothrombin time; INR, international normalized ratio. Bold values represented p < 0.05.

As shown in Table 1, the preoperative clinical characteristics were similar between two groups, except for that patients in the CRRT group were more likely to accompany malperfusion presentations including mesenteric ischemia (7.0 vs. 1.2%, *p* = 0.003) and coronary ischemia (7.0 vs. 1.2%, *p* = 0.003). For laboratory results, significant differences of c-reactive protein (CRP) (90.3 ± 70.9 mg/dL in the CRRT group vs. 70.7 ± 61.1 mg/dL in the non-CRRT group, *p* = 0.006), alanine transaminase, sCr, blood urea nitrogen, and fibrinogen levels were identified between two groups (Table 2).

### Surgical characteristics and outcomes

Our data showed that similar surgical techniques were applied in both groups. However, patients in the CRRT group had a significantly longer duration of cardiopulmonary bypass (CPB) (203.5 ± 117.0 min vs. 161.8 ± 58.8 min in the non-CRRT group, *p* = 0.006) (Table 3). In addition, patients in the CRRT group received increased amount of perioperative blood transfusion compared to those in the non-CRRT group (12.4 ± 3.9 units vs. 10.5 ± 4.1 units, *p* < 0.001).

**TABLE 3:** Comparison of operative variables.

Variables	Total (*n* = 503)	CRRT (*n* = 100)	Non-CRRT (*n* = 403)	*P*-value
Cannulation				
Femoral (%)	190 (37.8)	37 (37.0)	153 (38.0)	0.859
Axillary (%)	66 (13.1)	15 (15.0)	51 (12.7)	0.534
Axillary + femoral (%)	241 (47.9)	48 (48.0)	193 (47.9)	0.984
Concomitant CABG (%)	18 (3.6)	10 (10.0)	8 (2.0)	**0.001**
Concomitant MVR (%)	1 (0.2)	0 (0)	1 (0.2)	1.000
Concomitant MVP (%)	5 (1.0)	3 (3.0)	2 (0.5)	0.056
Concomitant TVP (%)	7 (1.4)	1 (1.0)	6 (1.5)	1.000
Concomitant RFA (%)	2 (0.4)	1 (1.0)	1 (0.3)	0.364
CPB time (min)	170.1 ± 75.9	203.5 ± 117.0	161.8 ± 58.8	**0.001**
Aortic cross-clamp time (min)	138.6 ± 37.5	137.6 ± 38.1	138.8 ± 37.3	0.762
DHCA time (min)	26.6 ± 14.2	28.4 ± 15.3	26.1 ± 13.9	0.151
Total arch replacement (%)	153 (30.4)	35 (35.0)	118 (29.3)	0.266
Operation time (h)	6.8 (5.8, 8.0)	7.6 (6.3, 9.0)	6.5 (5.7, 7.7)	**<0.001**
RBC transfusion (unit)	10.9 ± 4.1	12.4 ± 3.9	10.5 ± 4.1	**<0.001**

Values are mean ± standard deviation, median (interquartile range), or n (%).

CABG, coronary artery bypass graft; MVR, mitral valve replacement; MVP, mitral valvuloplasty; TVP, tricuspid valvuloplasty; RFA, radiofrequency atrial fibrillation ablation; CPB, cardiopulmonary bypass; DHCA, deep hypothermic circulatory arrest; RBC, red blood cell. Bold values represented p < 0.05.

Postoperative outcomes and major complications were summarized in Table 4. Unsurprisingly, the incidence of in-hospital complications was significantly increased in the CRRT group compared to the non-CRRT group (87.0% vs. 42.2%, *p* < 0.001). Compared with the non-CRRT group, the incidences of PMV, lung infection, and reintubation were significantly increased in the CRRT group. In addition, patients in the CRRT group had an 8.2 folds increase of 30-day mortality rate compared to the non-CRRT group (41.0% vs. 5.0%, *p* < 0.001).

**TABLE 4:** Comparison of postoperative variables.

Variables	Total (*n* = 503)	CRRT (*n* = 100)	Non-CRRT (*n* = 403)	*P*-value
AKI (%)	275 (54.8)	100 (100.0)	175 (43.5)	**<0.001**
Reintubation (%)	35 (7.0)	19 (19.2)	16 (4.0)	**<0.001**
Re-exploration for bleeding (%)	20 (4.0)	8 (8.0)	12 (3.0)	**0.028**
Tracheotomy (%)	22 (4.4)	10 (10.0)	12 (3.0)	**0.005**
Cerebral infarction (%)	47 (9.4)	31 (31.3)	16 (4.0)	**<0.001**
Cerebral hemorrhage (%)	4 (0.8)	4 (4.0)	0 (0)	**0.001**
Limb ischemia (%)	13 (2.6)	10 (10.1)	3 (0.7)	**<0.001**
PMV (%)	145 (29.2)	64 (65.3)	81 (20.3)	**<0.001**
Paraplegia (%)	22 (4.4)	12 (12.2)	10 (2.5)	**<0.001**
Gastrointestinal bleeding (%)	8 (1.6)	2 (2.0)	6 (1.5)	0.240
Lung infection (%)	202 (40.7)	73 (74.5)	129 (32.4)	**<0.001**
SWI (%)	8 (1.6)	3 (3.0)	5 (1.2)	0.367
Drainage in 24 h (mL)	1028.4 ± 819.6	1109.4 ± 796.8	968.3 ± 755.2	0.095
30-day mortality (%)	61 (12.1)	41 (41.0)	20 (5.0)	**<0.001**
ICU stay (day)	4.0 (1.0, 6.0)	10.0 (5.0, 19.0)	4.0 (1.0, 6.0)	**<0.001**
Hospital stay (day)	21.5 ± 12.1	24.7 ± 14.4	20.0 ± 9.8	**<0.001**

Values are mean ± standard deviation, median (interquartile range), or n (%).

AKI, acute kidney injury; PMV, prolonged mechanical ventilation; SWI, surgical site infection; ICU, intensive care unit. Bold values represented p < 0.05.

### Lactate

**FIGURE 2:**
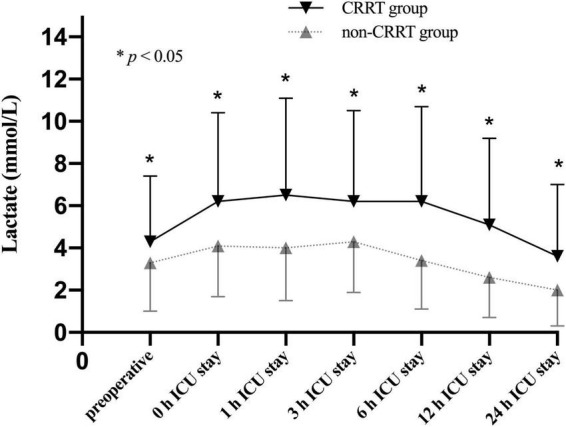
Lactate levels (mean and standard deviation) of patients in the CRRT group and the non-CRRT group at multiple time points.

**FIGURE 3:**
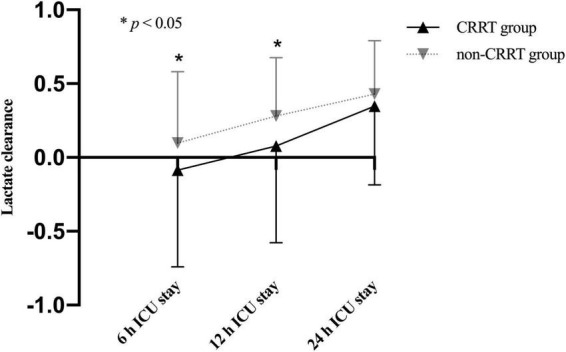
Lactate clearance (mean and standard deviation) of patients in the CRRT group and the non-CRRT group at multiple time points.

The average preoperative lactate level for overall cohort was 3.5 ± 2.8 mmol/L and presented with an upward trend upon ICU admission (4.5 ± 3.0 mmol/L) followed by a subsequent downward trend at 6 h after ICU admission (3.9 ± 3.1 mmol/L). Unsurprisingly, the perioperative lactate concentrations were significantly increased in the CRRT group compared to the non-CRRT group at all measurements (Figure 2). On the contrary, the lactate clearance was significantly decreased among patients in the CRRT group at 6 h (*p* = 0.010) and 12 h (*p* = 0.004) after ICU admission compared with the non-CRRT patients (Figure 3).

**FIGURE 4:**
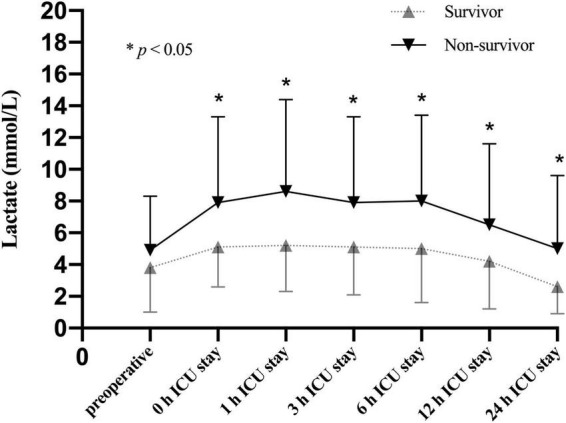
Lactate levels (mean and standard deviation) of patients stratified with 30-day mortality in the CRRT group at multiple time points.

**FIGURE 5:**
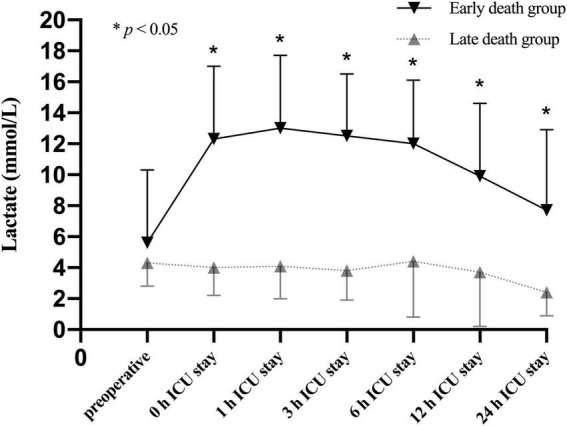
Lactate levels (mean and standard deviation) of patients stratified with the time distribution of death in the non-survivors of CRRT group at multiple time points.

To better understand the association between lactate levels and mortality, patients in the CRRT group were further stratified into survivors (59/100, 59.0%) and non-survivors (41/100, 41.0%) based on the 30-day mortality. Our results showed that the lactate levels were significantly increased in non-survivors compared to survivors only after operation (Figure 4). According to the time distribution of death, the non-survivors were divided into the early death group (death within 2–6 days after surgery, *n* = 20) and the late death group (death within 7–30 days after surgery, *n* = 21). Our data identified that the lactate levels after ICU admission were significantly elevated in the early death group compared to the late death group (Figure 5).

### Risk factors for receiving continuous renal replacement therapy

Next, we conducted a multivariable analysis to identify risk factors for receiving CRRT after ATAAD surgery and revealed that baseline sCr [odds ratio (OR), 1.008; *p* = 0.001], fibrinogen levels (OR, 0.657; *p* = 0.041), CRP (OR, 0.657; *p* = 0.041), CPB duration (OR, 1.007; *p* = 0.039), lactate level at 12 h after ICU admission (OR, 1.362; *p* = 0.039), and PMV (OR, 4.489; *p* = 0.002) were independent risk factors (Table 5).

**TABLE 5:** Multivariate analysis of risk factors for postoperative CRRT.

Variables	OR	95% CI	*P*-value
Preoperative sCr	1.008	1.003–1.013	**0.001**
Preoperative fibrinogen	0.657	0.440–0.983	**0.041**
Preoperative CRP	1.010	1.002–1.017	**0.009**
CPB	1.007	1.000–1.013	**0.039**
Lactate level 12 h after ICU admission	1.362	1.087–1.705	**0.007**
PMV	4.489	1.715–11.750	**0.002**

CRRT, continuous renal replacement therapy; OR, odds ratio; CI, confidence interval; sCr, serum creatinine; CRP, c-reactive protein; CPB, cardiopulmonary bypass; ICU, intensive care unit; PMV, prolonged mechanical ventilation.

Bold values represented p < 0.05.

### Risk factors for 30-day mortality in patients with continuous renal replacement therapy

As shown in Table 6, we further explored risk factors for 30-day mortality in patients with CRRT and the multivariable analysis showed that preoperative CRP (OR, 1.009; *p* = 0.021), increased lactate level upon ICU admission (OR, 1.284; *p* = 0.001), and decreased 24 h lactate clearance (OR, 0.237; *p* = 0.039) were independent predictors.

**TABLE 6:** Multivariate analysis of risk factors associated with 30-day mortality in patients with CRRT.

Variables	OR	95% CI	*P*-value
Preoperative CRP	1.009	1.001–1.018	**0.021**
Lactate level 0 h after ICU admission	1.284	1.107–1.489	**0.001**
24 h lactate clearance	0.237	0.061–0.928	**0.039**

CRRT, continuous renal replacement therapy; OR, odds ratio; CI, confidence interval; CRP, c-reactive protein; ICU, intensive care unit. Bold values represented p < 0.05.

### Receiver-operating characteristic analysis

**FIGURE 6:**
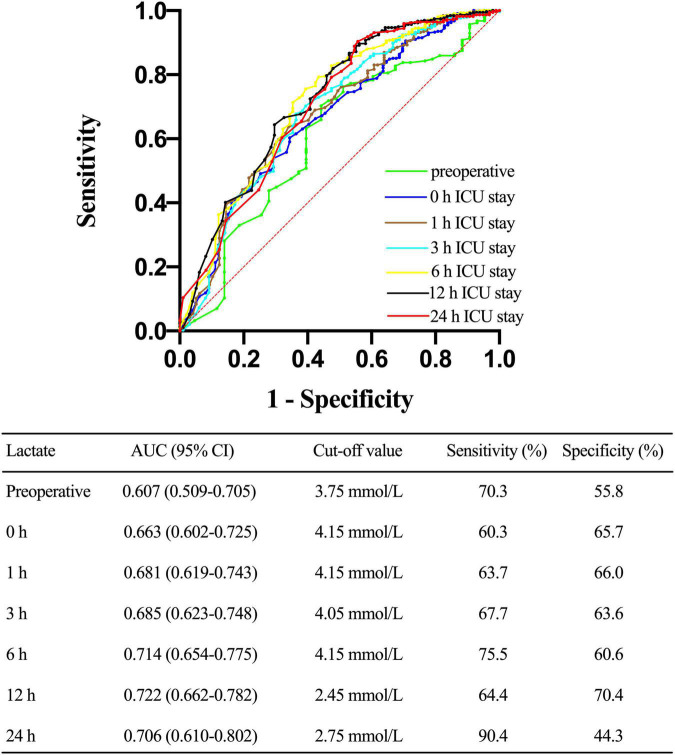
Receiver operating characteristic (ROC) curve comparing the ability of preoperative, 0, 1, 3, 6, 12, and 24 h lactate levels after ICU admission in predicting the requirement for continuous renal replacement therapy (CRRT) after acute type A aortic dissection (ATAAD) surgery. AUC, area under the curve; CI, confidence interval.

Next, we constructed a ROC curve to determine the predictive value of lactate concentrations on postoperative CRRT requirement. As shown in Figure 6, lactate levels of 4.15 mmol/L upon ICU admission, 4.15 mmol/L at 1 h after ICU admission, 4.05 mmol/L at 3 h of ICU admission, 4.15 mmol/L at 6 h of ICU admission, 2.45 mmol/L at 12 h of ICU admission, and 2.75 mmol/L at 24 h of ICU admission predicted the requirement for CRRT, with an AUC of 0.663 [95% confidence interval (CI), 0.602–0.725; *p* < 0.001], 0.681 (95% CI, 0.619–0.743; *p* < 0.001), 0.685 (95% CI, 0.623–0.748; *p* < 0.001), 0.714 (95% CI, 0.654–0.775; *p* < 0.001), 0.722 (95% CI, 0.662–0.782; *p* < 0.001), and 0.706 (95% CI, 0.610–0.802; *p* < 0.001), respectively.

Similarly, lactate clearance levels lower than 0.393 at 6 h of ICU admission and lower than 0.119 at 12 h of ICU admission predicted the requirement for postoperative CRRT, with an AUC of 0.591 (95% CI, 0.531–0.652; *p* = 0.005) and 0.585 (95% CI, 0.518–0.652; *p* = 0.034), respectively.

## Discussion

To the best of our knowledge, this study was the first to elucidate the associations between lactate concentrations and requirement of as well as prognosis of CRRT in patients who received ATAAD surgery. Our data showed that increased lactate levels at 12 h after ICU admission were associated with postoperative requirement of CRRT in ATAAD patients. Lactate levels at 6 h after ICU admission higher than 4.15 mmol/L and at 12 h higher than 2.45 mmol/L could be used to identify patients with CRRT after ATAAD surgical repair.

Previous studies suggested serum lactate could be used to predict mortality in patients who received cardiac surgeries (15, 16, 22). The lab-based prediction model including preoperative lactate, sCr, and liver malperfusion tests was proposed by Ghoreishi et al. (23) and has been externally validated recently (24). However, using lactate level measured at one time point could be misleading since it cannot accurately capture the ever-changing situations after surgery. More importantly, mild or moderate elevation are difficult to interpret at one single time point since they can go both up- and downwards in following disease course. Instead of a single lactate measurement, serial measurements or lactate clearance have been reported to be a more reliable indicator for prognosis in septicemia (19) and extracorporeal membrane oxygenation therapy (25). Our results were consistent with studies conducted in surgical and/or non-surgical settings which indicated that elevated serial levels of lactate and prolonged lactate clearance were associated with morbidity and death (26, 27). Polonen and associates (28) demonstrated in their study that restoring lactate levels decreased overall morbidity and total hospital stay in patients who received cardiac surgery.

Our data showed that the lactate concentration significantly increased after completion of CPB in all patients and further increased in patients who received CRRT. Multiple causes can contribute to the hyperlactatemia in perioperative period. Organ malperfusion is a severe complication may occur during aortic dissection and leads to severe ischemia. In addition, the capability of liver to clear lactate can be reduced secondary to the anesthetics and the low temperatures during CPB from a cellular metabolism perspective (29, 30). CPB may also tip the balance between oxygen demand and supply, which contribute to tissue hypoxia and even organ failure (31, 32). Furthermore, the use of catecholamines during cardiac surgery can increase lactate levels due to their effect on oxidative glucose metabolism (33). Last but not the least, stress-induced accelerated anaerobic metabolism after cardiac surgery can jeopardize tissue oxygen delivery, and, to a lesser degree, impair lactate clearance (17).

Patients who received complicated surgeries like ATAAD repairment often experience a transient postoperative hyperlactatemia that recover shortly. In our study, patients who had elevated lactate levels, such as higher than 3 mmol/L at 12 h and higher than 2 mmol/L at 24 h after ICU admission, might represent a subgroup of patients whose hyperlactatemia were secondary to occult tissue hypoperfusion. These findings suggested that clinical managements aim to reduce serum lactate levels to less than 3 mmol/L at 12 h and 2 mmol/L at 24 h of ICU admission might reduce the incidence of major complications including CRRT, as reported by previous studies (14, 34).

Unlike the findings reported by Maillet and colleagues (35) which showed that higher lactate levels at ICU admission were associated with increasing risks of developing major complications such as prolonged duration of mechanical ventilation and length of ICU stay. Our data demonstrated that the lactate level at 12 h after ICU admission, not the level upon admission, was independently associated with CRRT requirement after ATAAD surgery. We hypothesized that the hyperlactatemia observed at ICU admission may due to other etiologies than tissue hypoperfusion, such as cardioplegia, hypothermia, and CPB. However, persistent hyperlactatemia after ATAAD repair may indicate insufficient recovery of blood flow despite successful surgery, resulting in malperfusion syndromes. When CRRT was not avoidable, early initiation could improve outcomes. Our data showed that lactate levels higher than 2.45 mmol/L 12 h after ICU admission might help to guide the initiation of CRRT.

For patients who received CRRT, our study revealed that early lactate elevations after ICU admission and a reduced lactate clearance at 24 h after ICU admission were associated with increased 30-day mortality. Our results were consistent with a previous study focused on post cardiotomy patients which showed that the dynamic changes of lactate could be considered as a surrogate to predict early prognostic outcomes (36). Our results further confirmed the importance of restoring blood lactate levels within 24 h after ICU admission in patients who received ATAAD surgery.

Some conventional risk factors that have been known to be associated with worse outcomes after ATAAD surgery, including preoperative sCr, preoperative CRP, prolonged CPB times, and PMV also predicted the requirement for CRRT as suggested in our study and other previous studies (37). Compare to traditional indicators, the measurement of lactate has several advantages: rapidity, simplicity, convenient, and capability of repeated assessments. Adding lactate level to this prediction model might help physicians to better evaluate patients’ risk with an objective measurement and monitor the efficacy of treatment.

Although causes of increased high lactate levels might be vary, these results implicated the possibility of using lactate reducing therapy to prevent the use of CRRT after ATAAD surgery and increase overall prognosis.

## Limitations

This study had some limitations. All information was collected from patients’ medical reports and data was missing in some patients. All data was collected from one center and might not be representable to the general population. The sample size in some selected patient groups was relatively small. Moreover, this study was not powered to examine long-term prognosis.

## Conclusion

In conclusion, our results showed that dynamic lactate changes could be used to predict the need and outcome of CRRT in patients who received ATAAD surgery.

## Data availability statement

The raw data supporting the conclusions of this article will be made available by the authors, without undue reservation.

## Ethics statement

The studies involving human participants were reviewed and approved by the Affiliated Drum Tower Hospital, Medical School of Nanjing University, Nanjing. Written informed consent for participation was not required for this study in accordance with the national legislation and the institutional requirements.

## Author contributions

DW, ZW, and YK: research idea and study design. ZW, JX, LL, and LZ: data acquisition. ZW, JX, YK, and LL: statistical analysis. DW and ZW: supervision and mentorship. All authors contributed to the article and approved the submitted version.

## Abbreviations

ATAAD: acute type A aortic dissection
CRRT: continuous renal replacement therapy
ICU: intensive care unit
OR: odds ratio
sCr: serum creatinine
ROC: receiver-operating characteristic
CRP: c-reactive protein
CPB: cardiopulmonary bypass
AKI: acute kidney injury
PMV: prolonged mechanical ventilation.
